# Association of Cognitive Function, Quality of Life, and Sleep Disorders in Children With Depression

**DOI:** 10.62641/aep.v53i4.1866

**Published:** 2025-08-05

**Authors:** Li Xu, Yuewei Chen, Meili Liu, Huiwen He, Yanmei Shen, Jianhui Xie

**Affiliations:** ^1^Department of Neurology, The Affiliated Children's Hospital of Xiangya School of Medicine, Central South University (Hunan Children's Hospital), 410007 Changsha, Hunan, China; ^2^Department of Pediatrics, The Second Xiangya Hospital of Central South University, 410007 Changsha, Hunan, China; ^3^Department of Nursing, The Affiliated Children's Hospital of Xiangya School of Medicine, Central South University (Hunan Children's Hospital), 410007 Changsha, Hunan, China

**Keywords:** depressed children, sleep disorders, cognitive function, quality of life

## Abstract

**Background:**

Children with depression frequently experience sleep disorders, which may significantly impact their cognitive function and quality of life. Investigating the relationship between sleep quality, cognitive performance, and quality of life in this population is essential for developing targeted interventions.

**Methods:**

From February 2022 to January 2024, 78 children diagnosed with depression at the Hunan Children's Hospital were assessed using the 17-item Hamilton Rating Scale for Depression (HAMD-17). Based on their HAMD-17 scores, participants were categorized into mild, moderate, and severe depression groups, with 26 children in each group. Sleep quality was evaluated using the Pittsburgh Sleep Quality Index (PSQI), cognitive function was assessed via the Wisconsin Card Sorting Test (WCST), and quality of life was measured using the 36-item Short Form Health Survey (SF-36). Correlations between PSQI, WCST, and SF-36 scores were analyzed for all groups.

**Results:**

Compared to the control group, the depression group of children with depression had significantly higher levels of depression and significantly lower levels of quality of life, sleep quality, and cognitive function (*p* < 0.05). Further analysis showed that sleep quality in children with depression worsened with increasing severity of depression, as evidenced by a gradual increase in PSQI scores (*p* < 0.05). Cognitive function assessment (WCST scores) revealed that with increasing depression severity, the number of classifications completed by children decreased, while the total number of errors, perseverative errors, and non-perseverative errors all significantly increased (*p* < 0.001). Quality of life assessment (SF-36 scores) showed that increasing depressive symptoms significantly affected the quality of life of children, with an overall significant decrease in scores (*p* < 0.05). Correlation analysis further revealed that cognitive function was closely related to sleep quality in children with depression. Specifically, the number of classifications completed was significantly negatively correlated with PSQI scores (r = –0.5534, *p* < 0.0001), while the total number of errors, perseverative errors, and non-perseverative errors were all significantly positively correlated with PSQI scores (r = 0.6769, 0.6988, and 0.6937, respectively, all *p* < 0.0001). In addition, four dimensions of quality of life (social function, physical function, role function, and cognitive function) were all significantly negatively correlated with sleep quality (r = –0.6866, –0.5309, –0.5823, –0.5698, respectively, all *p* < 0.0001).

**Conclusion:**

Poor sleep quality in children with depression is positively correlated with poor cognitive function and poor quality of life. Routine evaluation of sleep disturbances in this population can provide critical insights for timely intervention and management.

## Introduction

Depression is a prevalent and severe mental health disorder that affects individuals across age groups, including children and adolescents [1, 2]. Childhood depression is characterized by a diverse range of symptoms that impact mental and emotional well-being. Depressed children often exhibit persistent sadness, feelings of helplessness and hopelessness, irritability, anxiety, fatigue, and significant mood swings. These symptoms contribute to difficulties in social interactions, academic performance, and family relationships compounding their challenges [3].

Cognitive abilities and quality of life are notably impaired in children with depression [4]. The instability of their mental state exacerbates depressive symptoms, creating a vicious cycle that hinders recovery and deepens emotional distress [5]. Cognitive deficits, including memory impairment and difficulty concentrating, are common in this population [6]. Such deficits make it challenging for them to learn new information, impede task completion, and negatively impact academic performance and self-confidence. Over time, these challenges can have long-term adverse effects on personal development and future opportunities. Moreover, depressed children experience a marked decline in quality of life, manifested by reduced daily activity capabilities, diminished interest in daily activities, and impaired social functioning [7]. These challenges often lead to social withdrawal and strained interpersonal relationships [8].

Sleep disorders are significantly prevalent among depressed children compared to their non-depressed peers [9]. Common sleep disorders include difficulty falling asleep, frequent nocturnal awakenings, early morning awakenings, and overall poor sleep quality [10]. Studies report that approximately 60–90% of children with depression experience varying degrees of sleep disorders [11]. A bidirectional relationship has been observed between sleep disorders and depressive symptoms. Poor sleep quality and insufficient sleep can exacerbate depressive symptoms, increasing emotional instability, irritability, and anxiety. Conversely, worsening depressive symptoms can further disrupt sleep patterns, creating a vicious cycle of poor sleep and deteriorating mental health [12].

Investigating the sleep quality of depressed children and its association with cognitive function and quality of life is of great clinical and theoretical significance. Sleep is essential for the physical, cognitive, and emotional development of children. Understanding the relationship between sleep disorders, cognitive functions, and reduced quality of life in depressed children can provide insights into the underlying mechanisms of depression, inform the development of targeted interventions, and establish a theoretical foundation for enhancing the overall well-being of affected children.

## Methods

### General Information

From February 2022 to January 2024, children and adolescents diagnosed with depression and treated at the Hunan Children’s Hospital were evaluated using the 17-item Hamilton Rating Scale for Depression (HAMD-17) [13]. Based on the HAMD-17 scores, each group consisted of 26 participants: mild depression (HAMD-17 score of 8-16), moderate depression (HAMD-17 score of 17-23), and severe depression (HAMD-17 score ≥24). The study cohort comprised 38 male and 40 female patients aged 9 to 18 (mean age: 12.71 ± 2.68 years). Additionally, 78 healthy controls matched by age and gender were recruited during the same period. The control group included 41 males and 37 females aged 9–18 (mean age: 12.79 ± 2.27 years). This study was approved by the Ethics Committee of Hunan Children’s Hospital (approval No.: HCHLL-2024-407), and all procedures adhered to the principles outlined in the Declaration of Helsinki. Informed consent was obtained from all participants and their guardians.

Inclusion criteria: (1) Individuals diagnosed with depressive disorder according to the International Classification of Diseases, 10th Revision (ICD-10) criteria [14]; (2) Individuals aged under 18 years and currently attending elementary school or having a higher educational level; (3) Individuals having no prior history of medication treatment; (4) Individuals willing to participate in the study; (5) Individuals with informed consent for participation provided by guardians.

Exclusion criteria: (1) Individuals without incomplete clinical data; (2) Individuals with other severe mental or organic disorders; (3) Individuals having mental disorders associated with substance abuse (e.g., drugs or alcohol); (4) Individuals having suicidal ideation or self-harm tendencies.

### Observational Indicators

(1) Sleep quality assessment: Sleep quality was evaluated using the Pittsburgh Sleep Quality Index (PSQI) [15]. The PSQI encompasses components such as sleep onset latency, sleep duration, daytime dysfunction, and subjective sleep quality. Each component is scored from 0 to 3, with the total PSQI scores ranging from 0 to 21. Higher scores indicate poorer sleep quality. A total PSQI score of ≥5 indicates a sleep disorder.

(2) Cognitive ability: Cognitive function was assessed using the Wisconsin Card Sorting Test (WCST) [16]. Participants completed the task on a computer using four stimulus cards and 128 response cards. Participants were required to match the response cards to stimulus cards based on rules such as shape or color, which changed without warning during the test. The task was performed on a computer, and the session ended after all 128 cards were used. The metrics recorded for analysis included total errors, completed classifications, non-perseverative errors, and perseverative errors. Higher numbers of completed classifications, coupled with lower counts for total errors, non-perseverative errors, and perseverative errors, indicated better cognitive performance. The total score was calculated as the sum of the total errors, completed classifications, non-perseverative errors, and perseverative errors.

(3) Quality of life: The 36-item Short Form Health Survey (SF-36) [17] was used to assess quality of life. The SF-36 evaluates four dimensions: social function, physical function, role function, and cognitive function. Scores for each dimension ranged from 0 to 100, with higher scores indicating a better quality of life.

### Statistical Analysis

Statistical analysis was conducted using SPSS 22.0 software (IBM Corp., Armonk, NY, USA). Data normality was assessed using the Shapiro-Wilk test. For comparisons between two groups, *t*-tests were used for normally distributed data, while the Mann-Whitney U test (rank sum test) was used for non-normally distributed data. For comparisons among multiple groups, one-way analysis of variance (ANOVA) was applied for normally distributed data, and the Kruskal-Wallis H test was used for non-normally distributed data. Spearman correlation analysis was employed to evaluate relationships between variables. Categorical data were expressed as frequencies and percentages [n (%)], and comparisons were made using the chi-square ($\chi^{2}$) test. A *p*-value < 0.05 was considered statistically significant.

## Results

### Baseline Data Comparison

Baseline characteristics between the depressed children and the healthy control group showed no significant differences in sex, age, or monthly household income (*p* > 0.05). However, the PSQI and HAMD scores were significantly higher in the depressed children group compared to the control group, while the SF-36 scores were significantly lower in the depressed group (*p* < 0.05, Table 1).

**Table 1.** **Comparison of general characteristics between healthy controls and children with depression [$\bar{x}$ ± s, M (Min, Max), n (%)]**.

Index		Control group	Depressed children	*t*/z/$\chi^{2}$	*p*-value
		(n = 78)	(n = 78)		
Gender	Male	41 (52.56%)	38 (48.72%)	0.23	0.63
	Female	37 (47.44%)	40 (51.28%)		
Age group (years)	9 ≤ Age < 12	30 (38.46%)	25 (32.05%)	2.80	0.25
	12 ≤ Age < 15	23 (29.49%)	33 (42.31%)		
	15 ≤ Age < 18	25 (32.05%)	20 (25.64%)		
Monthly household income (USD/month)	274.89 ≤ Income < 549.78	24 (30.77%)	28 (35.90%)	0.50	0.78
	549.78 ≤ Income < 824.67	28 (35.90%)	25 (32.05%)		
	824.67 ≤ Income < 1099.57	26 (33.33%)	25 (32.05%)		
HAMD-17 scores		4 (1, 6)	20 (8, 32)	10.83	<0.0001
PSQI scores		4 (0, 5)	6 (2, 9)	10.05	<0.0001
SF-36 scores		325.60 ± 24.97	246.18 ± 20.09	21.89	<0.0001

Note: HAMD-17, 17-item Hamilton Rating Scale for Depression; PSQI, Pittsburgh Sleep Quality Index; SF-36, 36-item Short Form Health Survey.

### Basic Characteristics of Children With Depression

The results showed no statistically significant differences in age, gender, and monthly household income among children with different degrees of depression (*p* < 0.05). See Table 2.

**Table 2.** **Basic characteristics of children with depression [M (Min, Max), n (%)]**.

Index		Mild group	Moderate group	Severe group	H/$\chi^{2}$	*p*-value
Age group (years)		13 (9, 17)	12.5 (9, 17)	12.5 (9, 17)	0.07	0.93
Gender	Male	12 (46.15%)	14 (51.85%)	12 (48.00%)	0.41	0.81
	Female	14 (53.85%)	12 (48.15%)	14 (52.00%)		
Monthly household income (USD/month)	274.89 ≤ Income < 549.78	10 (35.71%)	12 (42.86%)	6 (21.43%)	3.60	0.46
	549.78 ≤ Income < 824.67	9 (36.00%)	6 (24.00%)	10 (40.00%)		
	824.67 ≤ Income < 1099.57	7 (28.00%)	8 (32.00%)	10 (40.00%)		

Note: HAMD-17, 17-item Hamilton Rating Scale for Depression.

### Comparison of Sleep Quality in Children With Depression of Different Severity

The results showed no statistically significant differences in total sleep time among children with varying severity of depression (Kruskal-Wallis test, H = 2.27, *p* = 0.32). Pairwise comparisons with Dunn-Bonferroni correction revealed that, compared to the mild depression group, the severe depression group exhibited significantly reduced sleep efficiency (*p* = 0.001), elevated daytime dysfunction scores (*p* = 0.005), and worsened subjective sleep quality (*p* = 0.028). Additionally, when compared to the moderate depression group, the severe depression group demonstrated further declines in sleep efficiency (*p* = 0.005) and increased daytime dysfunction scores (*p* = 0.039), though the difference in subjective sleep quality was not statistically significant (*p* = 0.084). These findings indicate that the severity of sleep disturbances in children with depression intensifies with the progression of depressive symptoms, primarily manifested as progressively decreased sleep efficiency and continued worsening of daytime functional impairment (Table 3).

**Table 3.** **Comparison of PSQI scores in depressed children by severity [M (Min, Max)]**.

Group	n	Sleep time	Sleep efficiency	Daytime dysfunction	Subjective sleep quality
Mild group	26	2 (0, 3)	1 (0, 2)	1 (0, 2)	1 (0, 3)
Moderate group	26	2 (0, 3)	1 (0, 2)	1 (0, 2)	2 (0, 3)
Severe group	26	2 (0, 3)	1 (1, 2)^*#^	1 (0, 3)^*#^	2 (0, 3)^*^
H-value	/	2.27	16.13	11.06	7.83
*p*-value	/	0.32	<0.0001	0.004	0.02

Note: PSQI, Pittsburgh Sleep Quality Index. ^*^p < 0.05 versus mild group, ^#^*p* < 0.05 versus moderate group.

### Comparison of Cognitive Ability in Children With Depression of Different Severity

The WCST scores revealed significant cognitive impairments in children with moderate and severe depression compared to those with mild depression. Notably, the number of completed classifications decreased, while total errors, perseverative errors, and non-perseverative errors increased (*p* < 0.05). These findings indicate that cognitive abilities decline as the severity of depression increases. Furthermore, the severe depression group demonstrated significantly lower WCST scores compared to the moderate group, reflecting further deterioration in cognitive abilities (*p* < 0.05, Table 4).

**Table 4.** **Comparison of WCST scores in depressed children by severity [$\bar{x}$ ± s, M (Min, Max)]**.

Group	n	Classification completed	Total errors	Perseverative errors	Non-perseverative errors
Mild group	26	4 (3, 6)	42.69 ± 3.87	18.77 ± 1.78	9 (6, 12)
Moderate group	26	4 (2, 5)^*^	49.54 ± 4.34^*^	24.08 ± 1.74^*^	11 (8, 14)^*^
Severe group	26	3 (2, 4)^*#^	55.42 ± 5.04^*#^	26.62 ± 1.94^*#^	14 (10, 18)^*#^
F/H-value	/	29.31	53.55	125.73	57.11
*p*-value	/	<0.0001	<0.0001	<0.0001	<0.0001

Note: WCST, Wisconsin Card Sorting Test. ^*^p < 0.05 versus mild group, ^#^*p* < 0.05 versus moderate group.

### Comparison of Quality of Life in Children With Depression of Different Severity

SF-36 scores were significantly lower in the moderate and severe depression groups compared to the mild group, indicating a decline in quality of life with increasing depression severity (*p* < 0.05). Additionally, the severe group had significantly lower SF-36 scores than the moderate group, suggesting a further decline in quality of life (*p* < 0.05, Table 5).

**Table 5.** **Comparison of SF-36 scores in depressed children by severity [$\bar{x}$ ± s]**.

Group	n	Social function	Somatic function	Role function	Cognitive function
Mild group	26	65.19 ± 3.46	76.12 ± 6.80	57.92 ± 4.94	69.38 ± 4.90
Moderate group	26	59.85 ± 4.91^*^	66.73 ± 3.89^*^	54.31 ± 3.56^*^	64.12 ± 3.90^*^
Severe group	26	51.96 ± 6.14^*#^	62.81 ± 3.89^*#^	49.92 ± 2.90^*#^	60.23 ± 4.72^*#^
F-value	/	46.84	41.68	27.52	26.78
*p*-value	/	<0.0001	<0.0001	<0.0001	<0.0001

Note: SF-36, 36-item Short Form Health Survey; ^*^p < 0.05 versus mild group, ^#^*p* < 0.05 versus moderate group.

### Correlation Analysis of Sleep Quality With Cognitive Ability and Quality of Life in Depressed Children

**Fig. 1.**
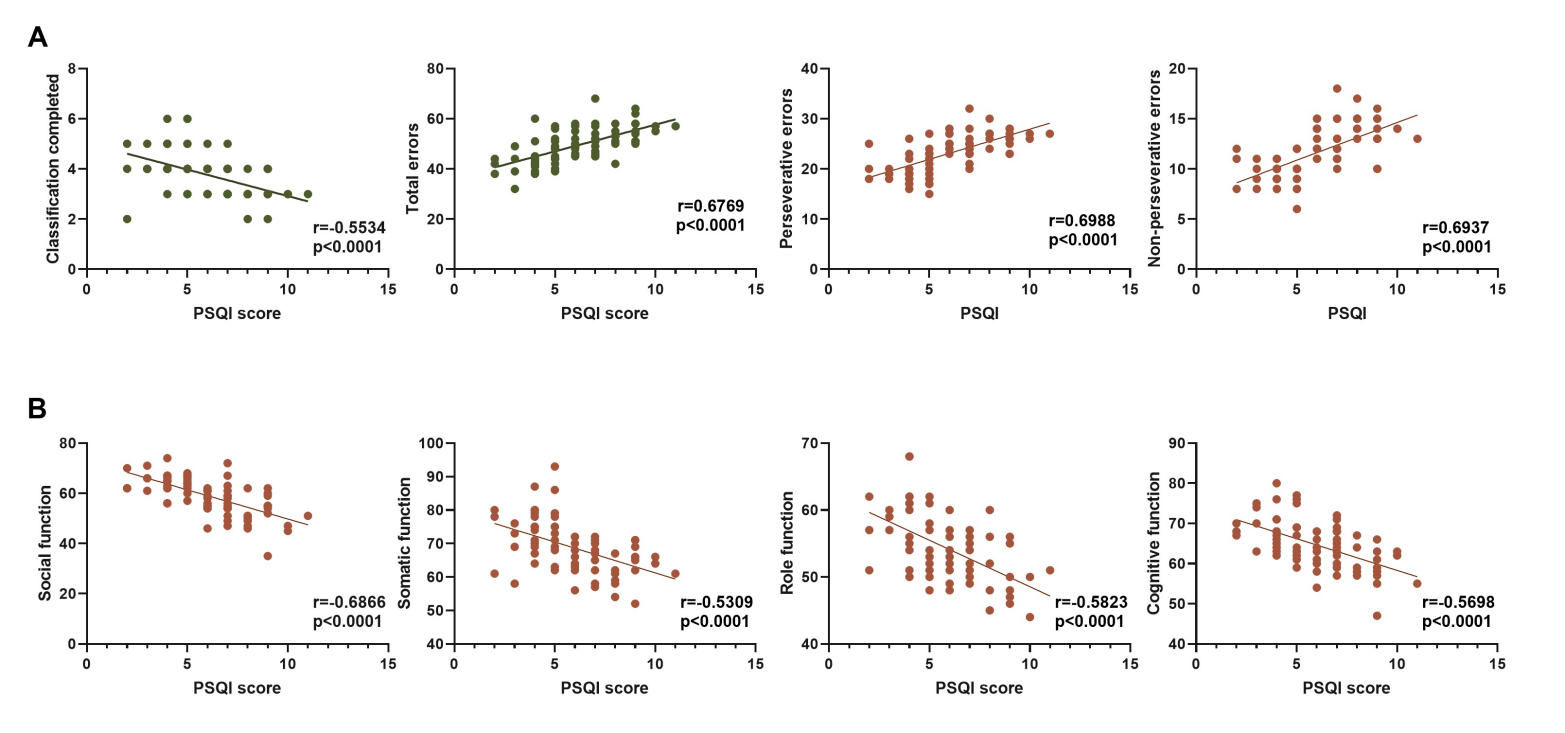
**Correlation between sleep quality, cognitive ability, and quality of life**. (A) Spearman correlation analysis between PSQI score and WCST score; (B) Spearman correlation analysis between PSQI score and SF-36 score. Note: PSQI, Pittsburgh Sleep Quality Index; WCST, Wisconsin Card Sorting Test; SF-36, 36-item Short Form Health Survey.

Spearman correlation analysis showed that PSQI scores in depressed children were negatively correlated with Classification completed in WCST scores (r = –0.5534, *p* < 0.0001) and positively correlated with Total errors, Perseverative errors, and Non-perseverative errors (r = 0.6769, 0.6988, 0.6937, respectively, all *p* < 0.0001). Additionally, PSQI scores were significantly negatively correlated with Social function, Somatic function, Role function, and Cognitive function in SF-36 scores (r = –0.6866, –0.5309, –0.5823, –0.5698, respectively, all *p* < 0.0001). These findings suggest that poor sleep quality is closely associated with cognitive impairment and decreased quality of life in children with depression (Table 6 and Fig. 1).

**Table 6.** **Correlation between PSQI scores and WCST scores, SF-36 scores in children with depression by severity**.

Index	PSQI scores	
	r	*p*
Classification completed	–0.5534	<0.0001
Total errors	0.6769	<0.0001
Perseverative errors	0.6988	<0.0001
Non-perseverative errors	0.6937	<0.0001
Social function	–0.6866	<0.0001
Somatic function	–0.5309	<0.0001
Role function	–0.5823	<0.0001
Cognitive function	–0.5698	<0.0001

## Discussion

Sleep disturbances are a common and significant challenge among children with depression. These disturbances often manifest as difficulty initiating or maintaining sleep and are both symptoms of depression and factors that exacerbate its severity [18]. Depressed children frequently exhibit symptoms such as low mood, poor concentration, and decreased daily functioning, which collectively impair their overall well-being [19, 20]. Investigating the interplay between sleep quality, cognitive abilities, and quality of life in children with depression is critical for developing comprehensive intervention strategies aimed at improving their overall health.

The findings of this study revealed that sleep quality in children with depression is significantly compromised and is strongly associated with the severity of depressive symptoms. Consistent with our findings, previous research has demonstrated a high prevalence of sleep disturbances in patients with depression, which, in turn, exacerbates depressive symptoms [21]. Notably, studies have increasingly highlighted the importance of sleep quality in managing pediatric depression. For instance, Üstündağ *et al*. [22] reported that poor sleep quality in children with depression exacerbates depressive symptoms. Similarly, research by Wichniak *et al*. [23] underscores that improving sleep quality can significantly alleviate depressive symptoms. These findings suggest that improving sleep quality is a key intervention for mitigating the severity of depression in children.

Pharmacological interventions targeting sleep disorders have also shown promise. For example, Moderie *et al*. [24] demonstrated that Quetiapine can improve sleep quality, thereby playing a role in treating insomnia associated with depression. Additionally, Jang *et al*. [25] demonstrated that Clonidine effectively enhances sleep quality in children and adolescents. These studies further underscore the pivotal role of sleep quality in the progression and management of childhood depression, emphasizing the need for deeper explorations of its association with the underlying pathophysiological mechanisms of depressive disorders.

This study also revealed that sleep quality, cognitive abilities, and quality of life decline as the severity of depression increases. Correlation analysis further revealed that cognitive function was closely related to sleep quality in children with depression. Specifically, the number of classifications completed was significantly negatively correlated with PSQI scores (r = –0.5534, *p* < 0.0001), while the total number of errors, perseverative errors, and non-perseverative errors were all significantly positively correlated with PSQI scores (r = 0.6769, 0.6988, and 0.6937, respectively, all *p* < 0.0001). In addition, four dimensions of quality of life (social function, physical function, role function, and cognitive function) were all significantly negatively correlated with sleep quality (r = –0.6866, –0.5309, –0.5823, –0.5698, respectively, all *p* < 0.0001). Research shows that children with depression commonly experience cognitive deficits, including inattention and memory decline, which adversely impact learning, social interactions, and daily functioning [19]. Furthermore, their quality of life is substantially affected, often characterized by reduced physical activity capacity, loss of interest in daily activities [20]. These challenges impact the immediate well-being of children with depression and may also have long-term adverse effects on their psychological and behavioral development.

The significant influence of sleep quality on cognitive abilities and quality of life in children with depression is further supported by existing literature. Sleep disorders are closely related to depression, and deteriorating sleep quality has been shown to exacerbate depressive symptoms [26]. Poor sleep quality is positively associated with cognitive decline [27], while emotional dysregulation due to sleep disturbances adversely affects social functioning and daily activities in children with depression [28]. These findings underscore the close association between sleep disorders, cognitive abilities, and quality of life in children with depression. Addressing sleep disorders may alleviate depressive symptoms and mitigate cognitive dysfunction and other pathological consequences in this population [29]. Further investigation into the interactions between sleep disorders and neural mechanisms in children with depression could uncover innovative treatment strategies for managing cognitive dysfunctions.

This study highlights a significant correlation between sleep quality, mental state, cognitive ability, and quality of life in children with depression. Improving sleep quality may positively impact the neurocognitive function and overall well-being of these patients. However, the study has several limitations. First, its cross-sectional design limits the ability to infer causal relationships. Second, the relatively small sample size may limit the generalizability and reliability of the findings. Future research should adopt longitudinal study designs to observe the long-term effects of sleep quality on cognitive function and quality of life in children with depression. Additionally, multicenter studies with larger cohorts, incorporating multidimensional assessments such as neuroimaging and biomarker analysis, are warranted to deepen our understanding of the relationship between sleep quality and the neurobiological mechanisms underlying childhood depression. These approaches could provide more robust evidence to inform clinical interventions and personalized therapies.

## Conclusion

Depressed children showed poor sleep quality, which was positively correlated with their poorer mental state, cognitive function, and quality of life. Regular assessment of sleep quality can offer valuable insights for timely management and intervention, thereby improving outcomes for this vulnerable population.

## Availability of Data and Materials

The data used and/or analyzed during the current study are available from the corresponding author.
